# Mobile-Based and Cloud-Based System for Self-management of People With Type 2 Diabetes: Development and Usability Evaluation

**DOI:** 10.2196/18167

**Published:** 2021-06-02

**Authors:** Raheleh Salari, Sharareh R Niakan Kalhori, Marjan GhaziSaeedi, Marjan Jeddi, Mahin Nazari, Farhad Fatehi

**Affiliations:** 1 Department of Health Information Management School of Allied Medical Sciences Tehran University of Medical Sciences Tehran Iran; 2 Endocrinology and Metabolism Research Center Shiraz University of Medical Sciences Shiraz Iran; 3 Department of Health Promotion and Education School of Health Shiraz University of Medical Sciences Shiraz Iran; 4 School of Psychological Sciences Monash University Melbourne Australia; 5 Centre for Health Services Research The University of Queensland Brisbane Australia

**Keywords:** type 2 diabetes, mobile health, mHealth, mobile app, self-management, behavior change

## Abstract

**Background:**

As the use of smartphones and mobile apps is increasing, mobile health (mHealth) can be used as a cost-effective option to provide behavioral interventions aimed at educating and promoting self-management for chronic diseases such as diabetes. Although many mobile software apps have been developed for this purpose, they usually lack a theoretical foundation and do not follow the guidelines suggested for evidence-based practice. Therefore, this study aimed to develop a theory-based self-management app for people with type 2 diabetes and provide an app based on a needs assessment analysis.

**Objective:**

This paper describes the development and usability evaluation of a cloud-based and mobile-based diabetes self-management app designed to help people with diabetes change their health behavior and also enable remote monitoring by health care providers.

**Methods:**

The development of this mHealth solution comprises 3 phases. Phase I: feature extraction of the Android apps that had a user rating of 4 stars or more and review of papers related to mHealth for diabetes self-management were performed followed by seeking expert opinions about the extracted features to determine the essential features of the app. Phase II: design and implementation included selecting which behavioral change and structural theories were to be applied the app and design of the website. Phase III: evaluation of the usability and user experience of the mobile app by people with diabetes and the portal by health care providers using the User Experience Questionnaire.

**Results:**

The developed mobile app includes modules that support several features. A person’s data were entered or collected and viewed in the form of graphs and tables. The theoretical foundation of behavioral intervention is the transtheoretical model. Users were able to receive customized messages based on the behavioral change preparation stage using the Kreuter algorithm. The clinician’s portal was used by health care providers to monitor the patients. The results of the usability evaluation revealed overall user satisfaction with the app.

**Conclusions:**

Mobile- and cloud-based systems may be an effective tool for facilitating the modification of self-management of chronic care. The results of this study showed that the usability of mobile- and cloud-based systems can be satisfactory and promising. Given that the study used a behavioral model, assessment of the effectiveness of behavior change over time requires further research with long-term follow-up.

## Introduction

Diabetes is a chronic condition in which the pancreas is unable to produce enough insulin to regulate glucose or the body cells cannot respond to insulin properly [1]. Diabetes is one of the most common metabolic causes of mortality because of its complications [2]. The prevalence of diabetes worldwide was estimated at 8.4% in 2017. This is projected to increase by 1.5% in 2045 [3]. The high prevalence of diabetes has high social and financial consequences, especially in low- and middle-income countries [3], including the Middle East and North Africa region [4]. The prevalence of diabetes in Iran is high and the number of affected people is continuously increasing. From 2005 to 2011, the prevalence of diabetes has grown by 35% in Iran [5-7]. The frequencies of diabetes-related complications among people with diabetes admitted to tertiary care centers in Iran are relatively high [7].

To minimize the potential risks of diabetes-related complications, patients should be educated and monitored to enhance their self-management abilities. People with diabetes who are empowered by self-management abilities show improvements in health outcomes [8]. People with diabetes must have access to ongoing health care services. However, in developing countries, financial and human resources are limited. Face-to-face education and self-management training for a person’s empowerment to control their illness are often suboptimal and limited [1]. Due to limited resources, the use of information technology has been suggested to improve a person’s self-management skills regularly. Although the use of the latest mobile technologies is valuable in empowering people with diabetes for self-management, health-related mobile apps are still relatively new and need more attention in developing countries [1].

The popularity of mobile health technologies has become an opportunity for education, remote monitoring, self-management, and data collection for diabetes care [9]. This opportunity can be used to provide interventions for user-centered and evidence-based self-management for people with diabetes [10]. Meanwhile, the development of these technologies, referred to as mHealth, has been greater than other apps [9]. mHealth is defined as “medical and public health measures supported by mobile devices, such as cell phones, personal monitoring devices, personal digital assistants, and other wireless devices” [11]. We have seen a large number of developed apps in the clinical care setting for patient self-management intervention [12], but few apps developed based on validated behavioral theories [13]. Interventions will have a greater chance of changing a person’s behavior if they use a behavioral model than interventions that have not used any behavioral model [14].

It is noteworthy that each behavioral theory can help us to understand why people behave concerning their health. Therefore, approaches that do not use these theories might fail [15]. Behavioral theory-based data collection for diabetes care can lead to customized feedback to enhance self-management skills. This feedback is a fundamental aspect of changing behavior that leads to improvement in a person’s self-management [9]. However, there is still no definite opinion on which behavioral models should be used to design an appropriate intervention for improving diabetes care [16]. Research shows that cognitive theories such as theory of planned behavior; transtheoretical model (TTM); and use of self-efficacy, information motivation, and behavioral skills were most widely used in technology-based interventions, especially mHealth apps [14].

One particular model provided by Prochaska and DiClemente [17] is the transtheoretical model. In this model, behavior change is considered as a 5-step process over time rather than an event. Since the results obtained from the use of this model have been reported to be useful for patients with type 2 diabetes mellitus (T2DM) [18], we decided to use this model in our study.

Two important points motivated the conduct of this study. In contrast to developed countries, in Iran, with the highest rate of diabetes prevalence in the Middle East and North Africa region [19,20], no study has been conducted to design a cloud- and mobile-based intervention underpinned by a behavioral model. Second, while most of the apps have been developed in the English language and there are already extensive studies on the usability of such apps [13], language remains one of the barriers in the adoption of English mobile apps by patients in non-English speaking countries [21]. There are numerous English apps in the public app stores for T2DM. Despite a large number of apps in this field, few of them are supported by scientific evidence, have been designed based on a behavioral model, and endorsed by health care professionals.

We felt the need for a Persian app for Iranian patients. However, we were not able to find any app in the Persian language that can provide management, monitoring, and education modules to Iranian patients based on a behavioral model. This paper reports on first Persian mobile app for diabetes care developed in Iran. In this study, we aimed to create a cloud- and mobile-based system for people with T2DM and health care providers to support diabetes self-management. We also evaluated the usability of the app. This paper sets out the steps taken to design and implement this app and highlights the limitations and results of the study. We believe the insight gained in this study illuminates the path for future studies in this field.

## Methods

### Study Overview

This study consisted of three main phases. To achieve a theory-based mobile- and cloud-based system that delivers a set of tailored messages, we first had to determine and define the requirements of such a system. One of these requirements was a behavioral model as a basis to design appropriate messages. Therefore, in the next phase, we determined which behavioral model we wanted to use according to the intended purpose of the system. The choice of model should be such that it can have the capacity to track patients over time and provide a suitable strategy for each stage of changing the patient’s care process. After that, the third phase was to design the mobile app and cloud-based system that included modules to meet the suggested requirements.

### Phase I: Needs Assessment and Planning

Initially, we conducted a survey to determine what features and functions are required for an ideal mobile app for people with T2DM [22]. We reviewed the literature and available diabetes apps for iOS and Android to investigate the features of diabetes apps to get familiar with them. The inclusion criteria were free apps directly related to diabetes with 4 or more stars (out of 5) in user ratings. Exclusion criteria were supporting only a single feature (for example, insulin calculation, recording data in an electronic notepad); not designed for diabetes self-management; provide information only such as how to use glucometer or educational materials about diabetes; not updated within 12 months prior to the search date; or solely targeting fitness, physical activity, or diet of people with diabetes. Relevant English articles indexed in Web of Knowledge, Google Scholar, PubMed, Scopus, and Science Direct published from 2012 to 2017 were retrieved using the keywords “diabetes,” “glucose,” “blood sugar,” “insulin,” “mobile Health,” “mobile apps,” “smartphone,” “mobile phone,” and “mHealth.” Articles that did not directly mention the use of mobile devices for the self-management of T2DM were excluded.

The validity of extracted features was then analyzed. For this task, we examined both the relevancy and necessity of features. We requested the members of 6 national interdisciplinary expert teams, including the Iranian Board of Health Informatics, Health Information Management, Endocrinology, and Health Education and Promotion, to provide comments on the most necessary and relevant features of a mobile app for diabetes management. Afterward, we designed a Likert-style questionnaire by verified features, to determine the final features list, and analyzed their level of importance (Multimedia Appendix 1). The response options to the questions ranged from 1=totally disagree to 5=totally agree. A total of 21 experts participated in the survey. The details of this phase have been reported elsewhere [22].

### Phase II: Design and Implementation

#### System Architecture and Model

A 3-tier model was applied as the conceptual model of the app. At the highest level, the logic layer presents the main functions of the app. This layer is responsible for processing the data and rendering them to the display layer. The data layer provides an interface to the logic layer and performs the necessary operations, including storing, editing, deleting, and retrieving data without engaging in the complexity of the database. In this layer, the database is designed and used. We needed to use a suitable model for customization of the message here. In the display layer, the application is placed under a web browser and a mobile app.

**Figure 1:**
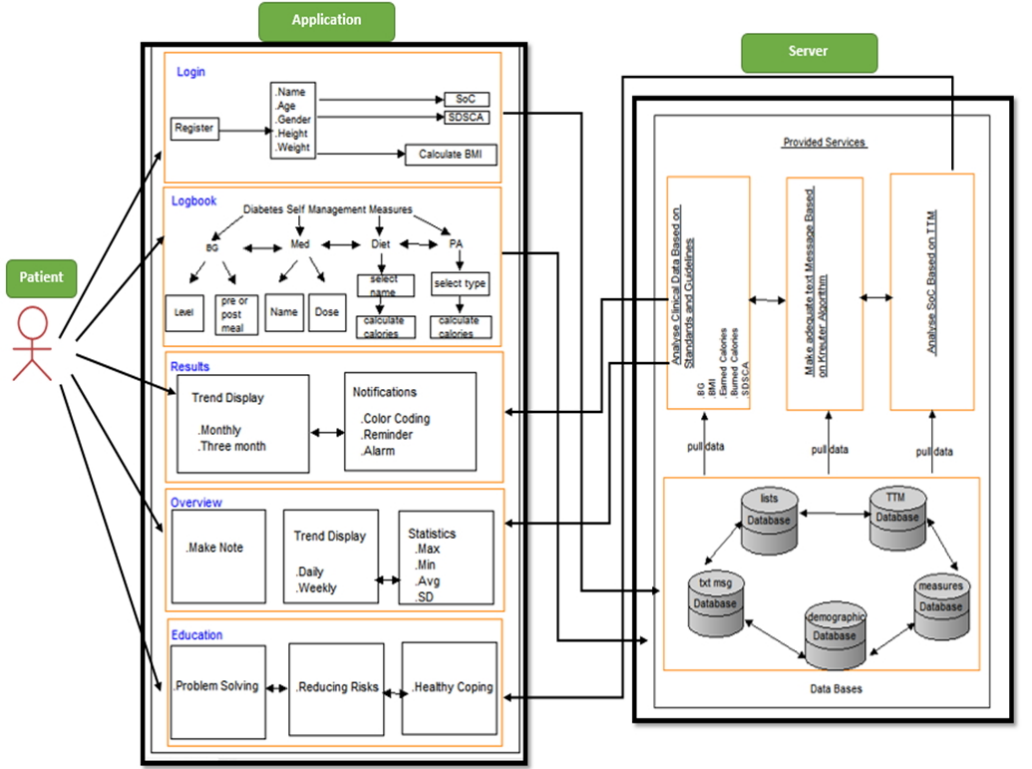
Proposed architectural framework for functionalities of a system for people with diabetes.

**Figure 2:**
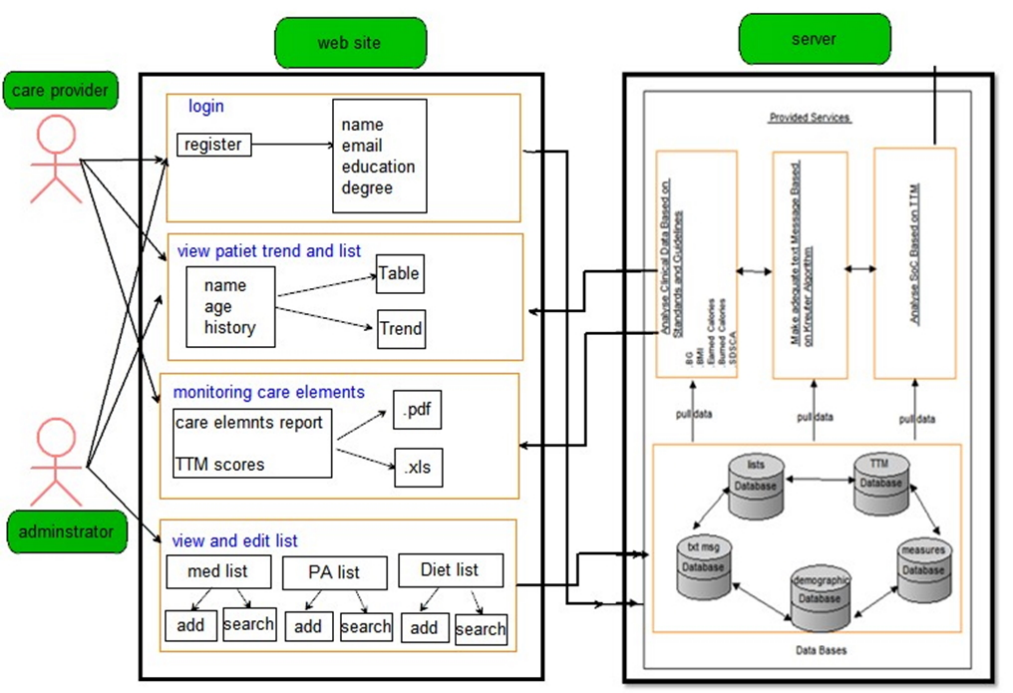
Proposed architectural framework for functionalities of a system for health care providers.

The mobile apps are designed for people with T2DM and health care providers. The users record the required data using the app and connected devices. Based on the status of the patient’s behavioral stage and other caregiving data such as blood glucose, physical activity, and calorie intake, message embedded in the library on the application server was called and displayed on the mobile phone screen. It is also possible to view care charts of the user at weekly, monthly, quarterly, and yearly intervals. The clinicians, on the other side, can monitor the patients’ data and be informed of their medication status, blood sugar, nutrition, and physical activity. The clinicians also can view notifications and messages sent to the patients. Figures 1 and 2 provide an overview of the framework designed for this cloud-based system and the mobile app.

The self-management app was designed to enable people with diabetes to manage their required care by monitoring blood glucose, physical activity, and diet as well as leverage the behavioral stage by receiving customized messages using smartphones. The app includes 5 modules that provide a platform for facilitating diabetes management, as suggested by American Diabetes Association. These modules include log-in and data management, logbook, analysis, overview, and education [23]. By module we mean a group of related features for performing a function in a mobile app. In this way, each module includes a set of features with specific tasks. For example, the overview module can provide an overview of the results of the care parameters over different periods, such as weeks, months, and years.

Many different theories guide health interventions. We explored and compared the existing behavioral models to identify the best model for further adaptation of the system according to the research goals and needs. To do this, the existing models were reviewed to reveal which model would help people with T2DM the most, and can be used for customizing messages for behavior change at a specified stage. TTM was identified as the best one for this purpose and formed the theoretical base of the app. TTM considers a person’s behavioral change in a chronic status as a multistep process [17]. In this case, the person’s behavioral stage status can be determined from the beginning (time of registration in the service) to the end (conclusion of the service). Staging of individual behaviors can be performed in two ways. One approach is to identify key behaviors based on their importance in diabetes management. They then implement step-by-step interventions for behavior change. The second approach is to focus on behavior that is almost ready for change [24]. In this study, we use the first approach, focusing on the important self-management behaviors of diabetes and monitoring the stages of change. These behavior aspects include physical activity, blood glucose monitoring, diet and nutrition, and medication, according to the International Diabetes Federation recommendations [25].

We also used the Kreuter algorithm, which was proposed in 1999, as a structured process to determine the customization of interventions. The reason for choosing this algorithm is to determine the level of intervention presented in this study that, based on a behavioral model, aims to deliver customized messages to help the self-management ability of diabetes persons [26]. The first step of the Kreuter algorithm is instrument identification and analysis. At this stage, a behavioral model is usually used as the baseline model to identify items for better diabetes care. In this study, the basic behavioral model for identifying the person’s problem is TTM. The second step of the algorithm is to use a tool or construct to evaluate the current condition of a person with T2DM by asking some questions about diet, medication, blood glucose measurement, and physical activity habits and behaviors. In the third step, messages were designed, and a library of messages was created. We chose those messages from publications that had developed the messages according to a scientific methodology. The messages were chosen according to their validity and relevance in the area of diabetes. To prepare a set of messages for this phase, we explored similar messages in past studies and existing educational resources and met several times with a health education specialist. We use the online training resources of the Iranian Ministry of Health and Medical Education [27], Association of Diabetes Care & Education Specialists [28], International Diabetes Federation [29], and American Diabetes Association [30]. In the fourth step of this algorithm, according to the previous step, the necessary responses were prepared and different modes were considered. In the final step of the algorithm, the rational rules for the relationship between the status and the appropriate message were made (ie, in other words, the relationship between the database and the library was designed).

#### System Modules

Before creating an app, it is necessary to provide a structure of each module to determine its functions and the relationship between these modules. To meet this need, we used diagrams to represent the structure and relationship of data elements and other components involved in each module and a scenario to illustrate the activities of that module. These diagrams are used during app analysis to identify requirements and illustrate how the app works. Actors in the mobile app and web application design include person with T2DM, specialist physicians, diabetes care nurses, and system administrators. Scenarios are the details of the set of commands or activities performed in a particular routine in an information system and include a sequence of operations that objects perform in the system.

As illustrated in Figure 1, a person with diabetes communicates by smartphone with the server. In this figure, the person with diabetes is the end user of the app, which encompasses 5 data modules. In Figure 2, on the server side, the databases include demographic information, data collection and analysis, drug and food list information, message and stage information, and the person’s stage according to TTM. The data for each database is called for its segment on which the relevant analysis is based. For example, a person’s data in addition to their stage of changes based on TTM is invoked and combined with the messages in the relevant database and displayed to them.

On the user side, the health care provider and the webmaster are the end users of the web portal, each with different levels of access. Moderator modules include log-in, access to medication list, physical activity, and food names. The manager has access to the list of health care providers. The health care provider has access to a list of people with diabetes and their care process, as well as a score based on the level of change, received and reported by TTM. Patients are scored in order of each stage of the change. For example, a patient who is in the first step of the physical activity does not even think about having an exercise program and gets a score of 1, and a patient who is in the fifth step of physical activity has been exercising for more than 6 months. On the server side, databases include demographic information, measurement and analysis information, drug and food list information, message information, and stage information according to TTM. The data for each database is called for its segment on which the relevant analysis is based. For example, data on monitoring a person’s information from their self-management process is called from the relevant database and displayed to the health care provider.

The use of the app begins with registration. The person enters the phone number (which triggers a confirmation code to be sent to the mobile number). The patient then enters a password and demographic information. A questionnaire based on TTM is presented in this section. The person completes the options for this questionnaire. The data from this section will be used to apply the rules of the Kreuter algorithm. People with diabetes should also include information about their weight, height, and level of physical activity. As mentioned earlier, these data will be used to calculate the number of calories needed and adjust their diet.

**Figure 3:**
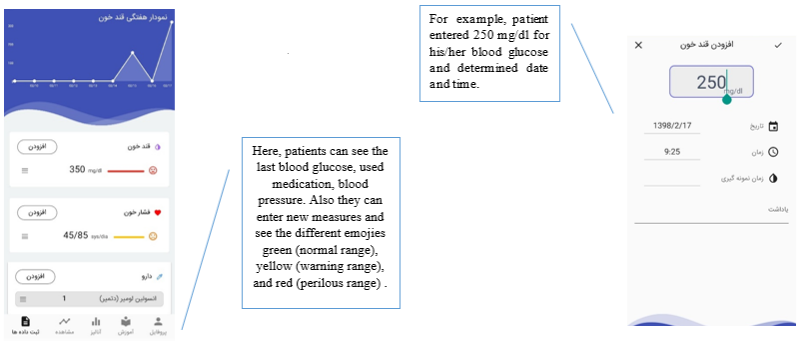
Logbook module where participants can enter their clinical data. This screen shows that the blood glucose level was entered and saved on a specific day and time.

In the logbook module (Figure 3), the person’s blood glucose levels, medications, diet, and physical activity should be entered. By selecting the 2-hour or fasting blood glucose (BG), the type of blood sugar measured will be recorded. In addition to receiving text messages, the app will display an emoticon that shows the patient’s blood sugar status in green (normal range), yellow (warning range), and red (danger range). Another parameter is the list of medications used. These medications are listed in alphabetical order, and pills and insulin can be selected from this list and the date and method of administration recorded. The next parameter is the database of foods that can be continuously updated. The app includes customized Iranian food databases for dietary intake. When a user exceeds the number of calories recommended for a day compared with the normal level and required calories based on the basic data, the person will receive a notification informing them to observe their diet. The food list is arranged alphabetically and allows people to determine the amount of food consumed. Depending on the type and amount of food consumed, the person’s intake calorie count will be calculated. For the physical activity, the person can select name and duration of their activities from the list, and their activity level will be calculated based on International Physical Activity Questionnaire.

**Figure 4:**
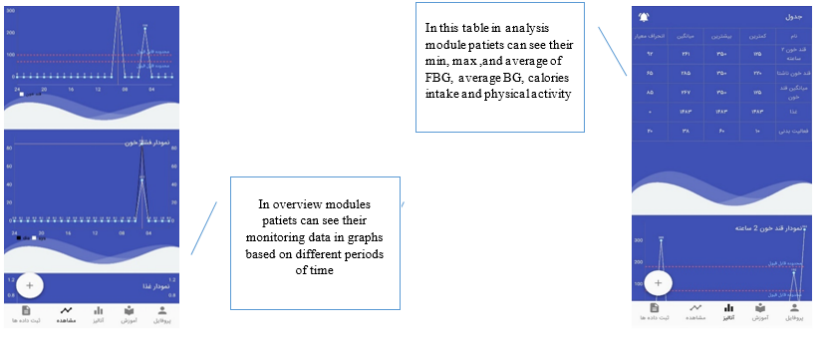
Overview and analysis module: participants can view the entered data in a chart format and see trends over time. Charts show physical activity range and calorie intake.

In the overview and analysis modules (Figure 4), users can see the daily and weekly charts of the 4 self-management aspects. There is also a graph to compare the calorie intake versus the number of calories consumed. Another feature of this module is that person’s data can be examined in a data table. In this table, we analyzed the statistical indices of mean, standard deviation, maximum and minimum blood sugar, calorie intake, and calories burned.

The data in the overview module can be explored in greater detail in the analysis module. In the analysis module of recorded data, monthly, quarterly, and annually surveyed comparisons are presented in linear graphs. The data comparison table of this module contains data in these time intervals. Another feature of this module is the ability of the people to define their own reminders. The user can set a specific time for a reminder during the process of self-management.

**Figure 5:**
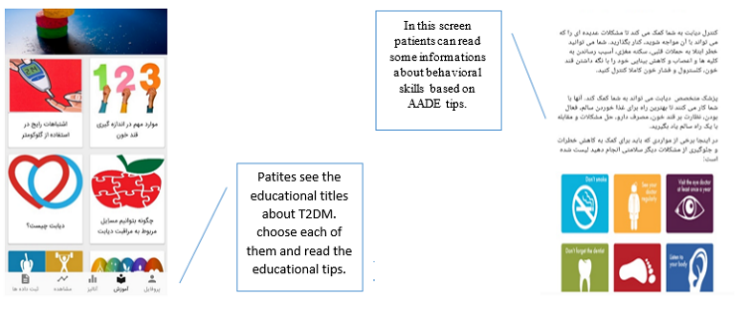
Education module: participants will learn tips on controlling diabetes, improving problem-solving skills, reducing risks, and healthy coping.

In the education module (Figure 5), users can receive general education and customized messages. The education module makes this information available to users regardless of the stage of change. The content of this module is also derived from the texts used to customized messages in addition to behavioral self-management recommendations, which are recommended by the Association of Diabetes Care & Education Specialists. This education includes tips on reducing risk, problem solving, healthy coping, and some general information about diabetes and using a glucometer.

For the health care providers, the web portal opens to a summary page that displays the person’s history and their trend in BG, diet and calories, physical activity, and behavioral stages. Selecting a person links to a web page displaying their contact details and details of their diabetes medication and expected number of BG recordings per week, month, and year. Once a person is selected, health care professionals can see a tabular display of BG readings that mimics a paper diary.

Graphical representations similar to the graphs displayed on the phone are also implemented on the web portal. The database, algorithms, and user interface for people with diabetes and health care providers were realized through coding. The developed app used the Android SDK platform 4.4.2 Java Development Kit. The database management program was developed using MYSQL. The app works on mobile phones running the Android operating system versions 2.3 to 4.4. The Laravel framework and the PHP programming language have been used to develop cloud-based software.

### Phase III: Usability Evaluation

The usability of the system and user satisfaction were assessed using health care providers and diabetes person’s data via using the User Experience Questionnaire (UEQ). UEQ is a standardized questionnaire in which end users describe their perception regarding aspects such as whether the app is easy to use, clear, confusing, and so on. This questionnaire measures 6 scales: efficiency, perspicuity, attractiveness, dependability, simulation, and novelty. The scales of the UEQ cover a comprehensive impression of user experience. Both classical usability aspects (efficiency, perspicuity, dependability) and user experience aspects (originality, stimulation) are measured. It consists of 26 contrarian adjective pairs randomly ordered to represent the 6 scales. The items are scaled from –3 to +3 with –3 representing the most negative answer, 0 a neutral answer, and +3 the most positive answer. All 14 recruited users evaluated the app for 10 days. This questionnaire has a useful tool developed in Excel (Microsoft Corp) that interprets the results and compares them with the results of previous studies in the same field.

Participation was voluntary, and the respondents could opt out of any phase of the study at any time. All participants were fully informed about the project. The identities of the participants were kept confidential throughout the process of data collection. The UEQ questionnaire was used to evaluate the app in the section based on the smartphone for people with diabetes and a cloud-based app for health care providers. We recruited 14 people with diabetes at the beginning, and all of them concluded the study. People were included if they met predefined criteria: aged over 18 years and under 60 years, have a smartphone with Android OS versions 2.3 to 4.4, not be insulin-dependent, at least 2 years have passed since they developed T2DM, be literate about using a smartphone, and be willing to participate in the study. The usability evaluation was conducted in Shahid Motahari Clinic of Shiraz and lasted for 10 days. Due to the limited number of health care providers (n=7), we did not consider any criteria and invited those who were interested in participating in the study to use and evaluate the intervention.

## Results

### Verified Features

The details of methods and results of the literature review as well as the process of selecting and verifying the features were published previously [22]. Based on expert opinions, 23 relevant features out of 33 were approved. These features were included: blood glucose, insulin and medication, physical activity, diet, weight and BMI, and blood pressure tracking; mealtime tagging; food database; educational materials; healthy coping; reducing risks; problem solving; messaging; color coding; alerts; reminders; target range setting; trend chart view; logbook view; numerical indicators view; customizable theme; preset notes; and custom notes.

### Testing the Cloud and Mobile-Based App

The mobile and cloud-based systems was then piloted in a usability evaluation by health care providers and people with T2DM. They were asked to use the app for 10 days and complete the UEQ questionnaire and give their feedback and suggestions.

In total, 14 patients (Table 1) and 7 health care providers took part in the study. The age range of patients was 24 to 53 years, most of them were female (10/14, 71%). The age range of health care providers was 35 to 42 years. Most of the health care providers who took part in this study were female (6/7, 85%).

**Table 1:** Results of the User Experience Questionnaire completed by patients (n=14).

Item	Median (SD)	IQR (Q3–Q1)
Attractiveness	1.56 (0.43)	0.56
Perspicuity	2.35 (0.82)	1.08
Efficiency	1.76 (0.50)	0.66
Dependability	1.70 (0.46)	0.56
Stimulation	1.96 (0.48)	0.64
Novelty	2.30 (0.60)	0.80

The highest median belongs to perspicuity and novelty and the lowest for attractiveness. The reason for the high perspicuity measure seems to be due to the simple design of the system. In designing this system, the features and modules were put together simply and clearly and enough explanations were written about each module for users. The point with the next highest measure is novelty. As mentioned in the previous sections of the paper, the existence and use of mobile-based systems in patient care and monitoring in Iran are still in its infancy. Therefore, when patients have used this system on their phones, its novelty seemed like one of the most prominent features. This measure has led to an increase in patients’ stimulation to use it.

The lowest level is related to the measure of attractiveness. Perhaps one of the main reasons for this is related to the simple design of the system. The textual content of the system instead of its visual content was applied. In other words, patients used this system by looking at the numbers in the charts, receiving recommendations and text messages, and selecting and typing the name of foods and physical activities in text form instead of images. Also, the lack of use of sound and music in the relevant sections could have reduced the attractiveness for patients. This content can increase the system attractiveness to users.

**Figure 6:**
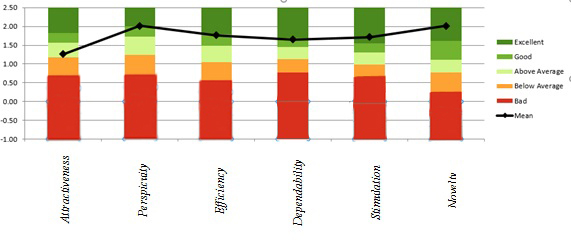
Comparison chart of average mobile app system measurements with previous studies.

The UEQ offers such a benchmark, which contains the data of previous product evaluations with these results. The benchmark classifies a product into 5 categories for each measure. Figure 6 illustrates this comparison for mobile-based system.

A total of 4 nurses and 3 physicians collaborated to evaluate the cloud-based app. Table 2 shows the median, interquartile range, and standard deviation of each measure asked among the health care providers. The highest average is for perspicuity and the lowest for attractiveness (Table 2).

**Table 2:** Results of the User Experience Questionnaire completed by health care providers (n=7).

Item	Median (SD)	IQR (Q3–Q1)
Attractiveness	1.53 (0.25)	0.32
Perspicuity	2.45 (0.62)	0.62
Efficiency	2.05 (0.34)	0.44
Dependability	2.15 (0.45)	0.62
Stimulation	1.95 (0.43)	0.60
Novelty	2.05 (0.30)	0.40

The highest scores were given for perspicuity. The reason for this is that we intended to design the system as clearly as possible for health care providers. We aimed to make it user-friendly so that working with it was not time-consuming. In addition, they were provided with sufficient explanations for each module. One of the reasons for the high dependability measure for health care providers could be the ability to view and access the data online and instantly. They could check the lists of educational recommendations and messages in addition to monitoring data and the process of changing stages of patients in the form of charts. In the usual state of care, it was not always been possible to observe the retrospective data in this way or it may have taken more time.

**Figure 7:**
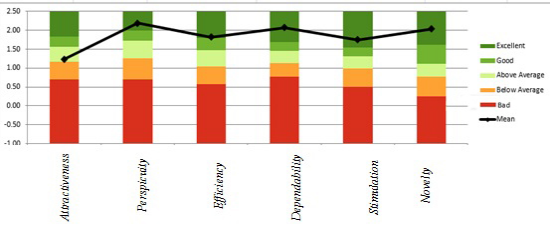
Comparison chart of average web-based system measurements with previous studies.

Similar to the results of the usability evaluation for patients, the lowest level of measurement is related to attractiveness. Perhaps one of the reasons for this is the simple design of the system. As mentioned, the appearance of the system was as simple as possible, so the use of images and a lot of colors was avoided. Figure 7 illustrates this comparison for the web-based system.

## Discussion

### Principal Findings

Using a cloud- and mobile-based system had many advantages for people with diabetes and health care providers. These apps could be helpful for persons receiving online education in self-management, relevant messages, and tips for their needs. Health care providers could also get an overview to conveniently obtain information about a person’s health and behavior status. Since cloud-based systems and mobile apps can be used as a tool for remotely monitoring and managing a person’s health, this feature can be effective in providing organized care for many other chronic diseases [1].

The app that we developed in this study differs from previous Iranian apps in a number of key aspects. It was developed based on a behavioral framework and provided customized messages and recommendations. Using this app, people with T2DM and their health care providers had multifunctional apps that enabled them to enter data in the logbook, view results in charts and tables, view them in more extensive time trends, receive generalized and customized messages and education, receive reminders and alarms, and have a concise useful clinical record. Apps that have multiple functions appear to be more likely to be used than those that have only a single function. The most common functions in diabetes apps include data documentation, data transfer, information collection, analysis, and reminders [31]. However, there must be a balance between the proper performance of an app and its user digital literacy level. The number and type of functions of the proposed app in this study were based on the applicable standards in the field and opinion of the specialists that we consulted.

Several studies have proven the effectiveness and benefits of charting data for diabetes care [32-35]. One of the features used in our proposed app was the use of charts to represent the data. Using visual approaches such as charts for blood sugar, physical activity, and calorie intake can be valuable to both people with diabetes and health care providers because they will be able to understand the information hidden in the data at a glance and make informed decisions better and faster by observing the process of change. Well-designed features in the mobile-based systems such as customized education demonstrated the potential for enhancing a person’s self-management outcomes. Developing such an app with customized feedback may help increase a person’s participation in telemedicine interventions as it provides relevant, timely, and specific feedback. Individual and behavioral characteristics of individuals are unique to them and can affect the level of self-management of each person differently. Therefore, tailored interventions can be more appropriate for each person’s individual needs [36].

In the study by Broderick and Haque [37], it was shown that there are 3 main barriers to mHealth interventions. These barriers include technical and human resources in organizations to support the implementation of these projects, lack of sufficient funding to finance investment in mobile technology solutions, and the challenges of merging health solutions with electronic health records and others. These barriers can also be compounded by user disinclination to cooperate and the fear of using tools, more common in developing countries. This can be attributed to a large number of specialist visits and the lack of sufficient time. People with diabetes themselves also acknowledge the concerns of using these apps, which may be related to their experiences of using unreliable online tools. This is a reasonable concern because few apps are research-based and even may be developed without any guideline-based or expert-approved content [22]. The results of this study showed that users were generally satisfied with the app. This overall positive feedback can be effective and promising for the first steps of implementing such apps in Iran. However, training on this issue and providing the right context can always be a challenge. Therefore, there should be more extensive research to respond to the growing needs under such circumstances. After this pilot study, further research is needed to investigate user attitudes and the likelihood of use in a larger population.

Since the researchers aimed to design and develop a new app rather than investigate the effects of using a predesigned system, the small number of users was not an obstacle to this goal. However, this number was really limited. Only 21 users (include 14 people with diabetes and 7 health care providers) used and evaluated this cloud- and mobile-based system, and the results cannot be generalized to all people diagnosed with T2DM. In other words, the purpose of this study was to design an mHealth intervention by using TTM, and the recruitment of potential users was required only for the initial deployment of the program. We performed only a usability evaluation in the real environment. The clinical outcomes of this intervention have not been studied yet and will be the topic of our next studies. Further clinical trial studies are needed to reveal the efficacy of this product compared with routine care.

### Conclusion

This study describes the development of a cloud-based and mobile-based system for people with diabetes and their health care providers. We used TTM as the theoretical foundation of this system and tailored massaging for improving the acceptability of the system by the users. Although some positive evaluation metrics were observed, a limited sample size did not allow for any concrete conclusions to be drawn from this study’s findings. More in-depth exploratory analysis of usability issues is needed to inform the design of clinical trials in this field.

## Appendix

Multimedia Appendix 1 Self-created questionnaire based on the transtheoretical model.

## Abbreviations

BG: blood glucose
mHealth: mobile health
TTM: transtheoretical model
T2DM: type 2 diabetes mellitus
UEQ: User Experience Questionnaire
