# Supplementary material for: Mobile-Based and Cloud-Based System for Self-management of People With Type 2 Diabetes: Development and Usability Evaluation
Source: J Med Internet Res. 2021 Jun 2;23(6):e18167. doi: 10.2196/18167 (PMC8209530; doi:10.2196/18167)
Supplement: Multimedia Appendix 1 [file jmir_v23i6e18167_app1.docx]

**The questions below ask you about your diabetes self-management activities. Please read carefully and select the most appropriate answer related to each question. The questionnaire is anonymous and your information will remain completely confidential to the researcher.**

| 1 | Given the definition of exercise and regular exercise, at least 3 sessions a week for 30 minutes each time, do you exercise regularly? |  |
| --- | --- | --- |
| A | No, Not yet. I haven't thought about that either |  |
| B | No, I have thought about it, but I haven't decided to do it yet. |  |
| C | No, now I have thought about this and I plan to exercise regularly for the next 30 days. |  |
| D | Yes, currently I have been exercising regularly for less than 6 months. |  |
| E | Yes, I have been exercising regularly for more than 6 months now. |  |
| 2 | Do you do this regularly, on average, at least 3 times a week, according to your regular blood sugar test? |  |
| A | Well, Not yet. I haven't thought about that either. |  |
| B | No, I have thought about it, but I haven't decided to do it yet. |  |
| C | No, I have thought about this and I plan to check regularly in the next 30 days. |  |
| D | Yes, currently I have been checking regularly for less than 6 months. |  |
| E | Yes, I have been checking regularly for more than 6 months now. |  |
| 3 | Do you take your medication as directed by your doctor? |  |
| A | Well, Not yet. I haven't thought about that either. |  |
| B | No, I have thought about it, but I haven't decided to do it yet. |  |
| C | No, I have thought about this and I plan to take it regularly in the next 30 days. |  |
| D | Yes, currently I have been taking it regularly for less than 6 months. |  |
| E | Yes, I have been taking it regularly for more than 6 months now. |  |
| 4 | Do you follow the necessary nutritional care under the supervision of a nutritionist or doctor regarding diet? |  |
| A | Well, Not yet. I haven't thought about that either. |  |
| B | No, I have thought about it, but I haven't decided to do it yet. |  |
| C | No, I have thought about this and I plan to follow it regularly for the next 30 days. |  |
| D | Currently, yes, I have been following it regularly for less than 6 months. |  |
| E | Yes, currently I have been following it regularly for more than 6 months. |  |
